# Unveiling and Benefits of Topically Applied l-(+)-Ergothioneine in Periwound Region †

**DOI:** 10.3390/ijms27146102

**Published:** 2026-07-08

**Authors:** Ladislav Šoltés, Anwar M. Jardine, Csaba Biró, Karol Švík, Mojmír Mach, Katarína Valachová

**Affiliations:** 1 Centre of Experimental Medicine, Slovak Academy of Sciences, 84104 Bratislava, Slovakia; ladislav.soltes@savba.sk (L.Š.); karol.svik@savba.sk (K.Š.); mojmir.mach@savba.sk (M.M.); 2 Faculty of Science, Department of Chemistry, University of Cape Town, Cape Town 7701, South Africa; anwar.jardine@uct.ac.za; 3 St. Elizabeth Cancer Institute Hospital, 81250 Bratislava, Slovakia; didymos75@centrum.sk

**Keywords:** chronic inflammation, HAT-hydrogen atom transfer, OCTN1 transporter, thione-thiol compounds, transdermal delivery

## Abstract

Oxidative stress significantly impairs wound healing by prolonging inflammation and damaging surrounding tissue. l-(+)-Ergothioneine (EGT), a naturally occurring thione–thiol antioxidant transported by OCTN1, exhibits potent cytoprotective and anti-inflammatory properties. The aim of the study was to investigate whether EGT applied topically to the periwound region can accelerate healing via transdermal antioxidant action. Full-thickness excisional wounds were created on the dorsum of Wistar rats. Animals were divided into three groups: the untreated rats (control), the rats treated with EGT at concentrations of 130 or 650 μg/cm^2^ applied on gauze positioned in the periwound region. Wound areas were measured over 15 days. Healing kinetics were analyzed using Logistic and Weibull models. The results showed that in the EGT-treated groups wound closures accelerated compared with controls. The Logistic model predicted ~ 70% healing in controls vs. > 90% in EGT-treated animals by day 15. The 130 μg/cm^2^ dose of EGT achieved near-maximal efficacy, while its higher loading had no substantial additional benefit. Topically applied EGT in the periwound region significantly enhances healing, supporting its role as a transdermally active antioxidant and a promising strategy for advanced wound care.

## 1. Introduction

The largest organ of the human body—the skin—is the primary integral barrier protecting the body’s structures from damage. The skin of an adult covers an area of approximately 1.5–2 m^2^, and its thickness ranges from 0.5 to 5 mm. The slightly acidic pH value of 4.0–6.0 ensures that the skin surface prevents the growth of bacteria, yeasts, and fungi [1]. The skin has several functions, including protection of the body from harmful substances, regulating temperature, providing sensory perception, and contributing to immunity. Since the skin is characterized by a thick (but permeable) membrane, numerous compounds can and do penetrate the skin, i.e., through the upper layer, the so-called *stratum corneum* (thickness 10–15 μm), to the lower layers, namely the *epidermis* (thickness 150 μm) and *dermis* (thickness 1–2 mm). Given the fact that the *dermis* is densely interwoven with blood vessels, low-molecular-weight compounds that have penetrated the lower layers of the skin—after entering the bloodstream—are distributed in the body. However, it is important to note that during the first cycle of blood circulation, exogenous compounds that have entered the bloodstream bypass the liver [2]. The skin, as a complex tissue, bleeds after injury and is stressed. Following injury, skin initiates an acute inflammatory response, progressing through proliferative and remodeling phases, leading to re-epithelialization. The formed scab falls off in a few days/weeks, and the injured area on the skin is healed. Nonetheless, in the case of chronic inflammation of injured skin, locally generated highly reactive oxygen/nitrogen species (ROS/RNS) can adversely contribute to wound healing and damage the surrounding healthy tissue so significantly that non-healing inflammation can result in the collapse of the organism [3].

Wound healing is a highly coordinated physiological process involving hemostasis, inflammation, proliferation, and tissue remodeling, all of which are essential for restoration of skin integrity. In pathological conditions, excessive oxidative stress, prolonged inflammation, impaired angiogenesis, and bacterial colonization can disrupt these processes and lead to chronic non-healing wounds. Current therapeutic approaches include hydrogel and polymeric wound dressings, antimicrobial agents, growth factor-based therapies, antioxidant-loaded biomaterials, and negative-pressure wound therapy. Experimental research in this field frequently employs the full-thickness skin excision wound model in rats, which is classified as a reproducible model for evaluating tissue regeneration and wound contraction. Recent studies have emphasized the importance of oxidative balance and advanced biomaterials in improving healing outcomes and reducing chronic inflammation associated with skin injury [4].

An important question arises whether topical antioxidants can mitigate oxidative stress and improve healing in the periwound region.

**Figure 1:**
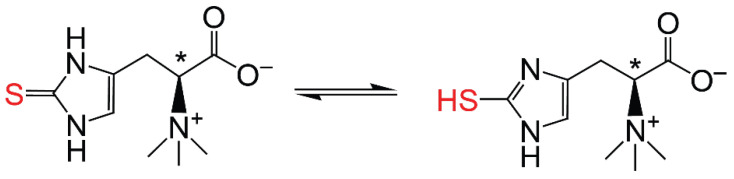
l-(+)-Ergothioneine is tautomeric; in neutral aqueous solutions exists predominantly in the thione form (**left**), which accounts for l-(+)-ergothioneine’s resistance to autoxidation. The thiol form (**right**) acts as a hydrogen atom transferring substance (HAT).

Several phylogenetically old organisms (genus *Actinomycetales mycobacteria* and non-yeast fungi—*Basidiomycota* and *Ascomycota*) [5] do not have the redox-active molecule l-glutathione (GSH), yet, as an analogous thione-thiol antioxidant, they have their own redox-active low-molecular-weight thiol antioxidant, namely l-(+)-ergothioneine (EGT). l-(+)-Ergothioneine, a derivative of thione-histidine-betaine, is in aqueous solution in tautomeric equilibrium [5,6], see Figure 1:

The human body does not synthesize EGT but has an active transport blood protein, namely the organic cation transporter (OCTN1) [7,8], with high specificity for the intake of EGT. Mammals acquire EGT solely through their diet. Foods such as mushrooms, black beans, red meat, and oats are rich in EGT. Because of its dietary origin, EGT represents a potential “vitamin” whose physiologic roles include cytoprotection [9,10,11,12,13,14]. OCTN1 expression is increased at any site of inflammation in the human body [15,16,17], especially in blood, lenses, liver, bone marrow, and seminal fluid, where EGT can reach millimolar concentrations. Patients with rheumatoid arthritis accumulate EGT in their synoviocytes. In bovine lenses, EGT concentrations of about 7 mmol/L exceed those of GSH 10-fold. In the bovine cornea, EGT concentrations are 14-fold higher than those of GSH, suggesting that it is the principal antioxidant in this tissue [18].

l-(+)-Ergothioneine scavenges ROS/RNS such as hydroxyl radicals, O_2_^•−^, and ONOO^−^. Moreover, it acts as a chelator of metal ions, namely iron and copper [19], and it is well-known for preventing DNA damage induced by ROS and RNS [20]. l-(+)-Ergothioneine protects skin cells from UV-induced oxidative damage by reducing the production of ROS, preserving reduced GSH, reducing mtDNA damage, and UV-induced MMP (matrix metalloproteinase) expression associated with photoaging [21]. Furthermore, EGT inhibits melanin synthesis, thereby reducing hyperpigmentation. It also has promising effects in repairing damaged collagen and elastin fibers, enhancing the skin’s moisture barrier, and reducing inflammation [11]. Commercial interest in EGT as an antioxidant has resulted in an explosion of products such as capsules containing this active principle. Yet, like all food, the liver’s enzymes metabolically process the capsules’ contents after absorption in the gastrointestinal tract.

To our knowledge, this is the first time the authors Valachová et al. [17] confirmed that l-(+)-ergothioneine incorporated into composite chitosan-hyaluronan membranes significantly accelerated the healing of ischemic skin wounds on rabbit ears compared to the control group (the untreated animals), as well as animals treated only with the control chitosan-hyaluronan membranes. However, subsequent pilot experiments in rabbits with the same content of EGT applied to the intact skin in the adjacent ear, instead of directly to the wound, were not entirely encouraging. Nevertheless, the anticipated outcome was obtained by increasing the loading of EGT applied in the periwound region.

**Figure 2:**
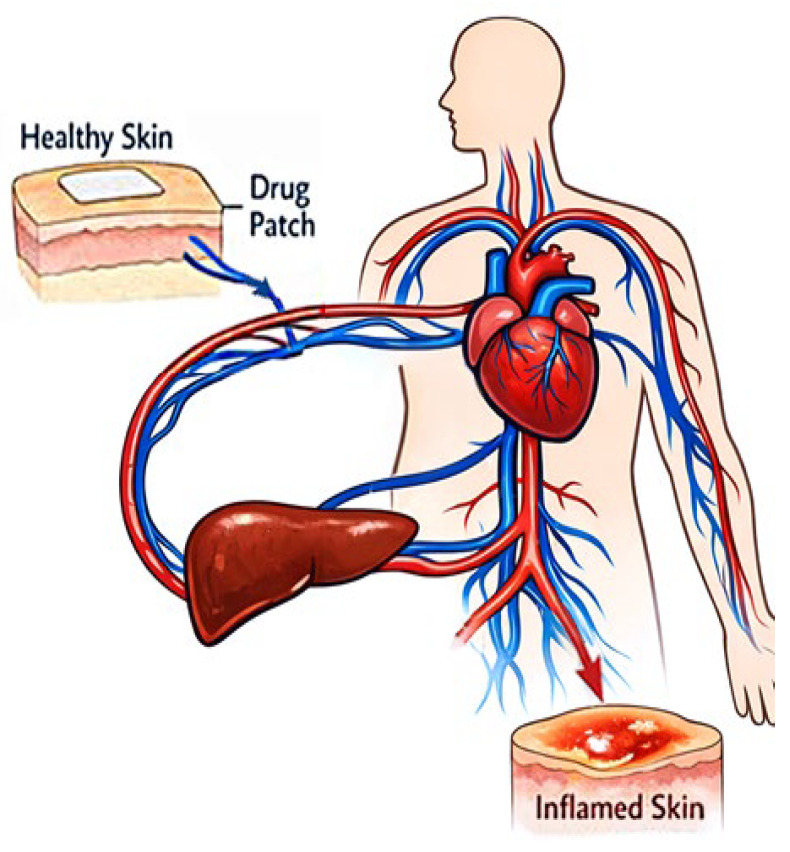
Illustration of the drug fate after the application onto healthy skin—the periwound region: (i) EGT is gradually released from its reservoir (the drug patch) after dissolution into sweat microdroplets formed on the skin surface. (ii) Subsequently, the aqueous EGT solution penetrates through the skin barrier and is absorbed into the bloodstream, where EGT is simultaneously complexed by the OCTN1 blood protein. (iii) The systemic blood circulation has the following steps: EGT is distributed throughout the body and extravasates from arterial capillaries into the inflamed skin tissue, i.e., the wound. Importantly, the site of EGT reservoir application is the periwound region, far from the site of inflammation, demonstrating the systemic transport of EGT from the healthy skin to the inflamed skin. (Figure 2 was generated using AI version 5.2 (https://chatgpt.com), accessed on 30 April 2026.)

The aim of this study is to describe in detail the experimental design and results demonstrating that topically applied l-(+)-ergothioneine may act as a transdermal antioxidant for treating skin injuries in the periwound region (cf. Figure 2).

## 2. Results

### 2.1. Monitoring of Wound Healing and Percentage of Wound Healing

**Figure 3:**
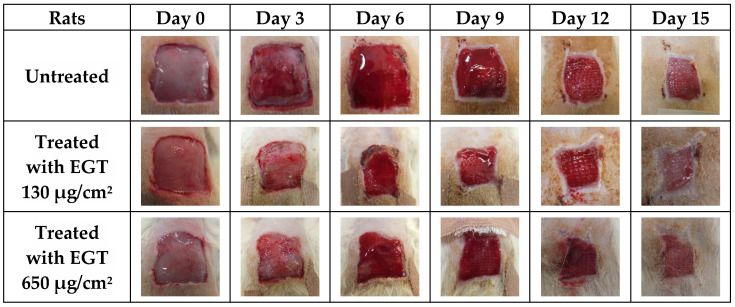
Process of closing the skin wound sized 2.0 × 2.0 cm of the control group (the untreated rat) and the rat treated with EGT at concentrations of 130 and 650 µg/cm^2^.

Based on the permissions (5465/2023-220; 2010/63/EU), we removed the skin of the rat on the back in the area of 2.0 × 2.0 cm, i.e., 4.0 cm^2^. The wound, in the control/untreated group of animals, closes during natural healing, while the sequence of physiological steps is characterized by the five following phases: hemostasis, inflammation, migration, proliferation, and finally maturation (remodeling), which leads to re-epithelialization. Figure 3 shows skin wound healing in the untreated and the treated rats on days 0, 3, 6, 9, 12, and 15.

**Figure 4:**
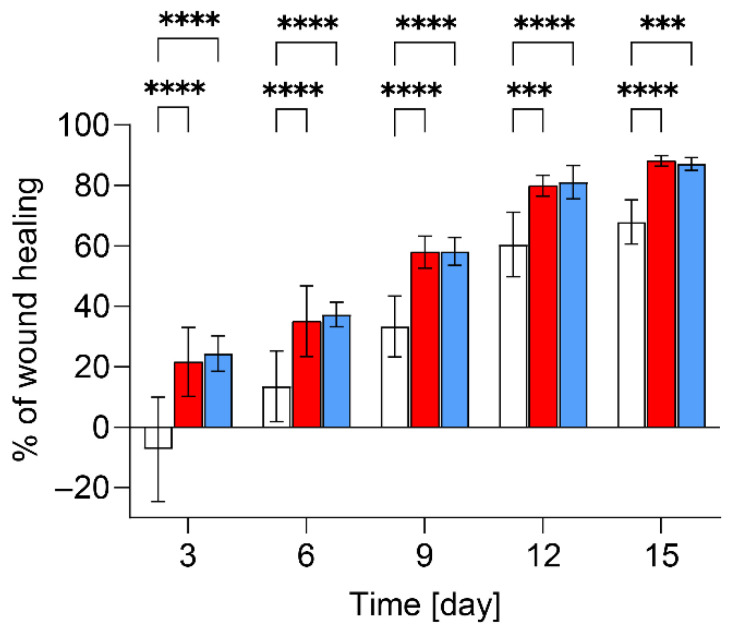
Time-dependent percentage of wound healing in the untreated rats (white column) and the rats treated with EGT at concentrations of 130 µg/cm^2^ (red column) or 650 µg/cm^2^ (blue column). Statistical analysis was performed using two-way ANOVA followed by Tukey’s multiple comparison test, *** *p* ≤ 0.001, **** *p* ≤ 0.0001 compared with the control group.

Results in Figure 3 and Figure 4 show faster contraction of wounds treated with either 130 or 650 μg/cm^2^ of EGT compared to the untreated rats. As shown in Figure 4, the percentage of wound healing in the untreated rats reached 67.9% on day 15 (white column). The addition of EGT in both doses resulted in faster wound healing from day 3, and on day 15 the percentage of healing was 88% (red and blue columns). Presentation of skin wound contractions in the control group and both groups of rats treated with EGT is in Figures S1–S3 in the Supplementary Materials.

**Figure 5:**
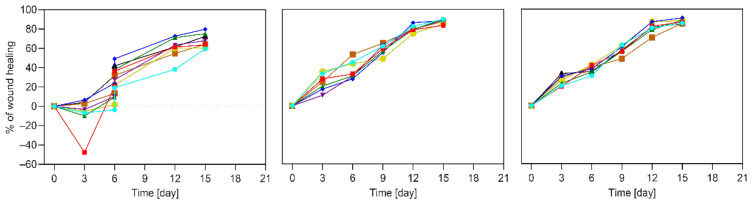
Time courses of wound healing in rats in the control group (**left panel**), in the group of rats treated with the gauze containing 130 μg/cm^2^ EGT in the periwound region (**middle panel**), and in the group of rats treated with the gauze containing 650 μg/cm^2^ EGT (**right panel**) in the periwound region. Each animal in the group is depicted in a different color.

As shown in the results presented in Figure 4 and Figure 5, in most control (untreated) animals (5 on day 3; 1 on day 6), the early enlargement of the wound area observed in the untreated control animals reflects the physiological inflammatory response associated with edema formation, vasodilation, and increased tissue tension [22]. In contrast, animals treated with EGT did not exhibit this pronounced early wound expansion, suggesting that EGT modulated the inflammatory response already during the initial phase of healing, during the first three days. This effect was evident as early as day three, when the treated wounds maintained a more stable wound area and showed earlier progression toward wound contraction. The findings support the hypothesis that transdermally delivered EGT attenuated excessive inflammatory dynamics before visible tissue regeneration occurred. Such modulation of the early inflammatory phase may represent one of the key mechanisms responsible for the accelerated healing observed in the EGT-treated groups.

In the group of animals with identical injuries, the treatment measure—namely the application of gauze containing 130 μg/cm^2^ EGT in the periwound region—resulted in practically immediate healing or wound closure. With this dose, we achieved a positive effect already on day three of healing; however, the values were still scattered.

For this reason, we performed an experiment with a megadose of EGT, the result of which is shown in Figure 5 (right panel). As expected, by applying the so-called mega-loading of EGT to the periwound region in the group of animals with the same injury, the scattering of data on healing was more uniform compared to the results represented in Figure 5 (middle panel). After three days in the group of animals with the application of 650 μg/cm^2^ EGT in the periwound region, the “gain” is uniformly 20.7−33.3%.

### 2.2. Histological Observations

**Figure 6:**
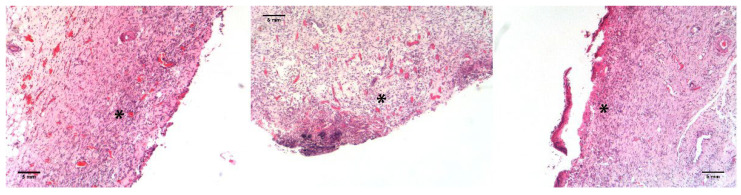
Histology of the untreated rats (**left panel**), rats treated with l-(+)-ergothioneine at a concentration of 130 μg/cm^2^ (**middle panel**) and 650 μg/cm^2^ (**right panel**). The asterisk represents the proliferation phase of wound healing.

Results of histology in Figure 6 showed that the tissues of the untreated animals, as well as tissues treated either with a lower or higher dose of EGT, were in the proliferative phase of healing on day 15.

### 2.3. Logistic Saturated Model of Skin Wound Healing

**Figure 7:**
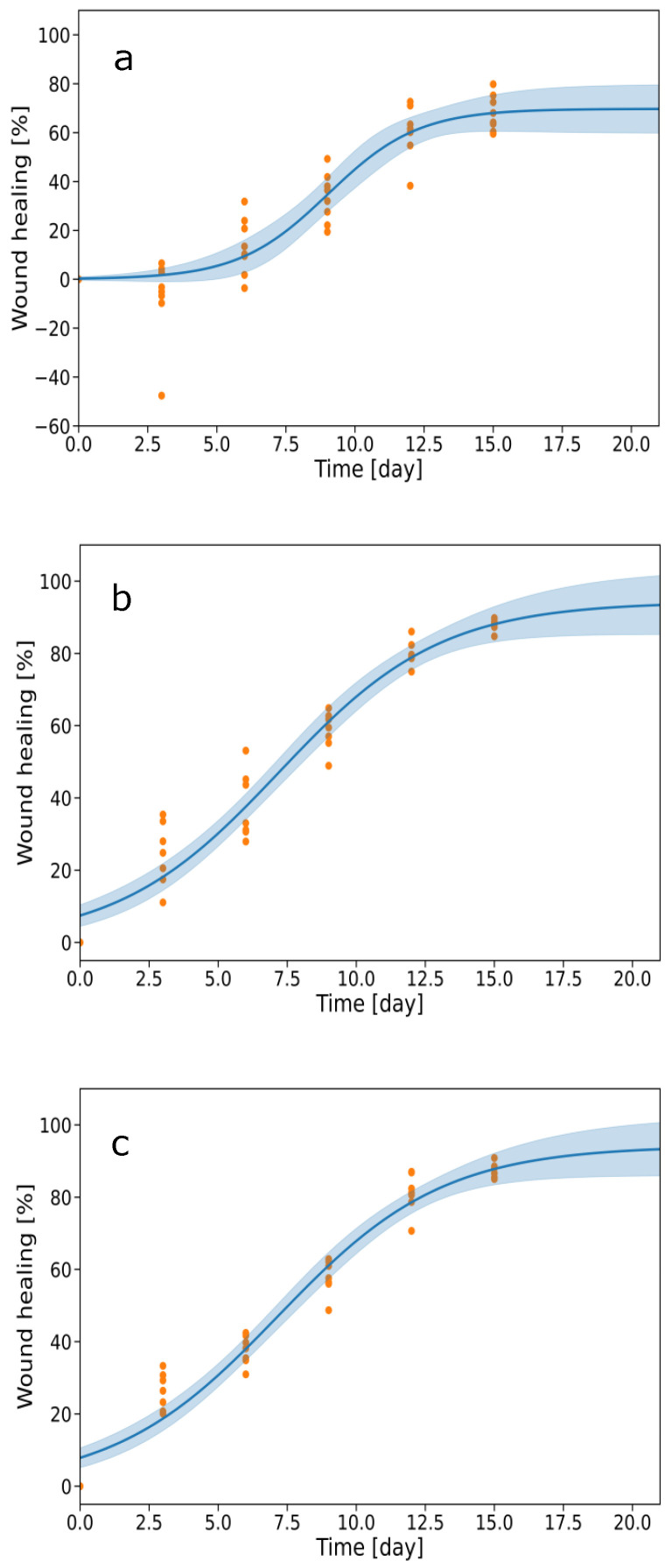
(**a**) Logistic model of wound healing with a 95% interval of confidence estimating the percentage of skin wound healing in the untreated rats, (**b**) in rats treated with l-ergothioneine at a concentration of 130 μg/cm^2^ and (**c**) in rats treated with l-ergothioneine at a concentration of 650 μg/cm^2^; over 15 days.

The observations in Figure 4 and Figure 5 underwent mathematical analysis using the Logistic model of wound healing as a first approximation. Figure 7 shows the comparison of the courses of time dependencies of healing wounds without EGT and loading EGT at two concentrations.

While, as evident in Figure 7, in the control group the dependent shape indicates that the treatment over 15 days, on average, is approx. 70%, in the treated animals the healing reached over 90% (EGT 130) and over 93% (EGT 650). Simultaneously, the treated groups’ interval confidence is narrower (cf. Figure 7c). As shown in Figure 7, the prediction of further improvement in healing is clearly evidenced by treatment with EGT, while in the untreated rats, the percentage of healing on day 15 and later is identical. Table 1 shows the kinetics of skin wound healing using the Logistic saturated model.

The results of the Logistic model in Table 1 show clear differences among the control group, EGT 130, and EGT 650 treatments. The asymptotic parameter L, which represents the maximum expected response, is substantially higher for EGT 130 (94.2%) and EGT 650 (94.2%) compared to the control group (69.7%). This indicates that both EGT treatments reach a markedly higher saturation level than the untreated control.

The growth rate parameter k is highest in the control group, while it is lower and very similar for both EGT 130 and EGT 650. This suggests that although the control group increases more steeply around its inflection point, it ultimately saturates at a lower maximum value.

The inflection point x_0_, representing the time (or x-value) at which the growth rate is maximal, occurs later in the control group (9.03) compared to EGT 130 (7.21) and EGT 650 (7.17). Thus, the EGT treatments reach their rapid growth phase earlier than the control group.

The standard error of estimate (s≈) is lower for EGT 130 and EGT 650 than for the control group, indicating a better model fit for the treated variants.

The Logistic model suggests that the treatment with EGT at both concentrations accelerated the response, shifted the inflection point to earlier x-values, and substantially increased the maximum achievable level compared to the control. The Weibull model is described in detail in the Supplementary Materials (cf. Figure S4, Table S1).

## 3. Discussion

Nature possesses intricate mechanisms to keep living systems healthy. Among the defense systems, GSH functions as a key antioxidant in phylogenetically younger organisms, where it prevalently donates a hydrogen atom to neutralize oxidants and becomes oxidized to glutathione disulfide (GSSG). The enzyme glutathione disulfide reductase regenerates GSH from GSSG, maintaining redox balance and cellular protection [23,24]. 

In contrast, phylogenetically older organisms, exposed primarily to cosmic radiation rather than the present high concentration of atmospheric oxygen, evolved to use EGT as a principal protective compound. Current research suggests that EGT, a naturally occurring thiol derivative of histidine, exhibits strong antioxidant, cytoprotective, and anti-inflammatory properties, making it a promising candidate for enhancing wound healing processes [25].

Wound healing is a complex, five-step process involving multiple cell types, such as fibroblasts, endothelial cells, immune cells, and soluble mediators. Disruption of these interactions leads to chronic wounds, requiring sustained intervention for at least three weeks or until measurable tissue recovery is observed [26]. Animal models remain a key experimental approach for evaluating healing efficacy, with variables including species selection, wound size, and location. Skin wounds are the most common wounds, usually caused by the carelessness of the injured individuals. In case of scientific research, variables such as animal species, location, and extent of the wound need to be considered.

Prior studies have demonstrated successful healing outcomes using chitosan/hyaluronan-based membranes loaded with active agents such as edaravone, MitoQ, and l-glutathione. To our knowledge, the efficacy of EGT has been for the first time examined when loaded into a skin wound dressing, which we submitted as a patent application [14]. The invention concerned self-associating biopolymers comprising polyanionic (high-molecular-weight hyaluronan) and polycationic (chitosan) biopolymers, which together form an artificial skin carrying the cytoprotectant—l-(+)-ergothioneine—loosely incorporated into the carrier. The results of this research were subsequently published by Valachová et al. [17]. Since in the membrane l-(+)-ergothioneine can be trapped by negatively charged hyaluronan, it is superior to use only a neutral gauze, enabling simple release of l-(+)-ergothioneine.

Also, in our previous studies, we experimented with two animal species, particularly the rat and the rabbit. The location of the wound in rats was the dorsal area, and the size of the wound was always 1.5 × 1.5 cm, i.e., 2.25 cm^2^, and in ischemic rabbits the size of the wound was 1 × 1 cm, i.e., 1 cm^2^ [27]. However, in this study, based on the project with the approval no. 5465/2023-220, the wound size in rats was even 4 cm^2^. Notably, the loading of EGT at a concentration of 130 μg/cm^2^ proved to be appropriate for healing acceleration, whereas EGT at a higher concentration (650 μg/cm^2^) did not provide additional benefit, suggesting a dose-dependent plateau.

According to the Lipinski Rule of Five [28], a compound’s ability to penetrate the dermal barrier depends on molecular weight, polarity, and solubility. l-(+)-Ergothioneine appears capable of penetrating the dermis following topical application, demonstrating that a water-soluble, positively charged molecule can access deeper tissue. The proposed mechanism involves OCTN1 transporters, which facilitate EGT uptake into systemic circulation and target inflamed tissues, where it acts as a HAT antioxidant.

A particularly important observation of this study was the prevention of the initial wound expansion in EGT-treated animals, which was otherwise evident in the untreated controls during the first days after injury. Since early wound enlargement is typically associated with acute inflammatory reactions, including edema and marginal tissue stress, the inhibitory effect of EGT strongly indicates an early anti-inflammatory action mediated through transdermal penetration. This observation suggests that EGT may regulate inflammatory signaling before the onset of intensive proliferative repair, thereby creating more favorable conditions for subsequent tissue regeneration. The ability to attenuate excessive inflammatory dynamics without completely suppressing physiological inflammation may explain why EGT-treated wounds achieved faster wound closure. These findings further support the concept of EGT as a transdermally active cytoprotective antioxidant with therapeutic potential in advanced wound-healing strategies.

However, the extended half-life of EGT (~1 month) [18] raises concerns regarding systemic accumulation during repeated topical application, potentially altering its local pharmacodynamics during acute inflammation.

**Figure 8:**
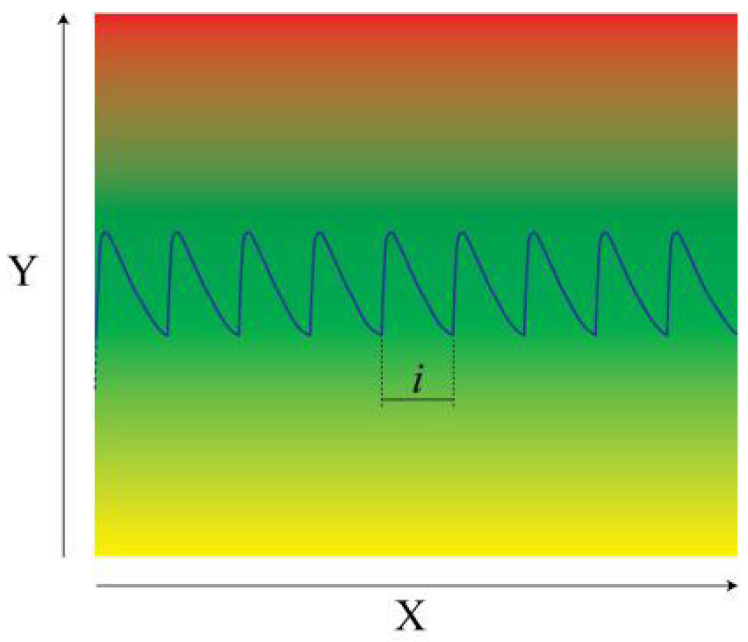
The concentration of a drug or a medicinal substance (blue) (axis Y) during the period of treatment (axis X) must be in a range of effective treatment levels of the drug or of the medicinal substance (area marked in green), whereas it must always avoid the range of toxic levels (area marked in red), and should avoid the range of therapeutically ineffective levels of the drug or of the medicinal substance (area marked in yellow). The time interval between two consecutive applications of a gauze is indicated—***i***.

As depicted in Figure 8, the topical EGT administration via gauze mimics a controlled, slow infusion system. After each application, EGT concentration decreases below therapeutic levels within approximately three days, necessitating replacement to maintain effective exposure. This dosing model ensures therapeutic efficacy while minimizing toxicity.

From the above, it is possible to directly deduce that the peak of the blue wavy line is the EGT concentration on the skin immediately after the gauze is applied to healthy skin. The gradual decrease in EGT in the gauze in the time interval ***i*** can be expressed by several mathematical equations. And since the instantaneous EGT concentration at the point of contact gradually decreases, it can be implicitly stated that the same trend of EGT decrease is present in the skin. This phenomenon indicates that wound tightening is functionally dependent on the absorption and subsequent penetration of EGT from the point of contact into the blood/venous capillaries, as well as on the bioavailability of EGT within the wound itself, regardless of its distance from the site where the medicinal substance is applied.

As evident in Figure 4, the untreated wounds initially expanded during the first three days, while EGT at a concentration of 130 μg/cm^2^-treated wounds resisted expansion and achieved 87.2% healing on day 15, compared to 68% in untreated controls. These results align with predictions from Logistic and Weibull modeling, affirming EGT’s sustained healing effect and supportive potential as a therapeutic antioxidant.

Collectively, these findings support EGT’s dual role as both a redox regulator and a biological enhancer of tissue regeneration. The compound demonstrates promising applicability in the development of biocompatible wound dressings, with the added benefit of naturally occurring biochemistry and low cytotoxicity risk. The observed dose-dependent saturation effect also provides a valuable insight into formulation optimization for clinical translation.

## 4. Materials and Methods

### 4.1. Materials

l-(+)-Ergothioneine was obtained from Tetrahedron, Paris, France. The gauze roll (width 10 cm, thick-woven, 0.1 mm thick, very soft, extremely absorbable, breathable, and long-lasting) was obtained from Tri M Medical, Cairo, Egypt. Novasul^®^ was purchased from Richter-Pharma AG, Wels, Austria. Zoletil^®^ and Shotapen^®^ were purchased from Virbac S.A., Carros, France. Hematoxilin & eosine, 10% formalin, and xylene were purchased from Sigma-Aldrich, Darmstadt, Germany.

In compliance with Directive 2010/63/EU, the pilot study adheres to the directives of the State Veterinary and Food Administration of the Slovak Republic, Bratislava (5465/2023-220) and the Ethics Committee of CEM of the Slovak Academy of Sciences in Bratislava, Slovakia.

### 4.2. Preparation of Gauzes Impregnated with l-(+)-Ergothioneine

The concentration of the EGT stock solution was 9 mg/mL. The appropriately diluted aqueous EGT solution was applied dropwise onto a dry Egyptian cotton gauze within a marked area (13 × 7.7 cm; 100.1 cm^2^) placed on a polyterephthalate (PTF) plastic pad (Figure S5A). Owing to the high absorbency of the gauze, the entire EGT solution was absorbed within 5 min, allowing the wetted gauze to adhere firmly to the PTF pad without curling. The pad carrying the EGT-impregnated gauze (Figure S5C) was frozen at −20 °C for 24 h and then immediately transferred to the bottom shelf of an LL3000 lyophilizer (Trigon Plus, Ltd., Čestlice, Czech Republic) for freeze-drying over 24 h. After lyophilization, the dried gauze was removed from the PTF pad, and it was confirmed that no visible white EGT residue remained, indicating essentially complete retention of EGT within the gauze (Figure S5E). The amount of incorporated EGT was determined gravimetrically by subtracting the weight of the untreated gauze from that of the EGT-loaded gauze. The net EGT loading was 13.1 mg (130.9 µg/cm^2^) and 65.1 mg (650.3 µg/cm^2^) per 100.1 cm^2^ of gauze for the low- and high-dose preparations, respectively.

### 4.3. In Vivo Experiments in Rats

For the experiments, 22 male 10–12-week-old outbred Wistar rats (weight 270 ± 10 g) used were from the Department of Toxicology and Laboratory Animal Breeding, CEM, Slovak Academy of Sciences, Dobrá Voda, Slovakia. The study was performed based on the project with approval no. 5465/2023-220 on 11 September 2022. After acclimatization (5 days), the rats were kept in a 12 h dark/12 h light cycle and had unlimited access to food and water according to the EU Convention for the Protection of Vertebrates Used for Experimental and Other Purposes. The control group included eight animals; the groups of animals treated with EGT each comprised seven. A schematic presentation of the in vivo study design is depicted in Figures S6 and S7 in the Supplementary Materials.

Excisional wounds were created using a modified induction method based on the standard model described by Frank and Kämpfer [29]. An excisional wound to the skin tissue of size 2 × 2 cm was created. We applied and fixed a gauze (2 × 2 cm) containing EGT at loads of 130 and 650 µg/cm^2^ to the shaved area of skin (the periwound region), 1 cm away from the wound in the treated animals. Sterile Sterilux^®^ (SteriLux, Renens, Switzerland) was applied and fixed to the damaged area. The rats received an intramuscular antibiotic Shotapen^®^ (0.1 mL/kg) for the next three days. After the procedures, the animals were provided with standard postoperative care. To prevent pain, the rats were given an analgesic Novasul^®^ (0.1 mL/kg, i.m.) immediately after induction and then daily throughout the study. The gauzes with the incorporated EGT were changed at regular intervals on days 3, 6, 9, and 12. On days 0, 3, 6, 9, 12, and 15 of the study, physical indicators such as live weight were monitored by weighing the rats, and the area of the wounds was measured with a digital caliper to determine the acceleration of wound healing. Subsequently, the macroscopic morphology of the damaged tissue changes was evaluated based on photographs obtained using a digital camera. On day 15, the experiment finished, and the animals were killed after administering the anesthetic Zoletil^®^ (100 mg/kg, i.m.).

### 4.4. Histology

The identity of the histological sections was blinded during the analysis. After the experiment, the tissues fixed in 10% formalin and dehydrated and cleared in xylene were embedded in paraffin to form blocks, which were cut into 5.0 µm sections, transferred to slides, and stained with the most commonly used staining—hematoxylin and eosin [30]. Morphological indicators of skin healing were evaluated, based on which we determined lesions without healing (the inflammatory phase), lesions with partial healing (the proliferative phase), and completely healed lesions (the remodeling phase).

### 4.5. Mathematical Modeling of Wound Healing Kinetics

For the mathematical evaluation of the kinetics of skin-rat wound healing, we used the Logistic saturated model, which is expressed with the following equation:

y = L/[1 + e^[−k(X − x_0_)]]

L: Maximal effect of healing.
k: Speed of healing/day (the higher the value, the faster the speed).
x_0_: Breaking point (where it is 50% of L).

For comparison with the Logistic saturated model, in the Supplementary Materials we used the Weibull saturated model limited to 100% healing (cf. Figure S4, Table S1 in the Supplementary Materials), which is expressed by the following equation:

y = L [1−e^−(x/t)^m^]

L: Maximal effect of healing equals to 100%.
t: Time scale (when the effect begins to appear).
m: Steepness (how sharply the curve breaks).
ν: Degrees of freedom.

## 5. Commentary

In the dermis, OCTN1 binds EGT and transports it through the bloodstream to the site of injury and bypasses the liver in the first cycle. It can suppress chronic inflammation, thereby shortening the inflammatory phase. This allows for a reduction in the overall healing time; however, it is not indefinite because the phase in which inflammation is necessary must first be completed. l-(+)-Ergothioneine, even at the lower concentration (130 µg/cm^2^), was remarkably effective in healing skin wounds in rats. It is obvious that wound healing cannot be accelerated even with the higher EGT loading. Our preliminary findings suggest that each of the five phases of healing, such as hemostasis, inflammation, migration, proliferation, and remodeling, requires a certain time to reach a particular level of wound healing. Even in the lower concentration of EGT (130 µg/cm^2^), the healing process proceeded in the fastest possible way.

l-(+)-Ergothioneine’s sulfhydryl group underpins its HAT antioxidant properties, scavenging reactive oxygen, nitrogen, and superoxide anion radical species, thus protecting cells from a variety of apoptotic insults. Moreover, it inhibits TNF-α-induced release of the inflammatory IL-8 in alveolar macrophages [5].

## 6. Conclusions

l-(+)-Ergothioneine incorporated into gauze demonstrated significant transdermal antioxidant activity, accelerating wound closure and promoting organized tissue regeneration in rats. These findings support its potential usage in advanced wound dressings and broader dermatological applications. Because the gauze is applied only to healthy tissue, sterility is not strictly required. Moreover, during dressing changes, the healing site remains undisturbed.

Further translational research is needed to define the optimal clinical use of EGT and to determine whether it should be considered a compound with broader systemic benefits. Although OCTN1 has been proposed to transport l-(+)-ergothioneine to sites of inflammation throughout the body, the rate of transdermal transport—especially when using penetration enhancers—remains to be clarified. Structural modification of the molecule may ultimately improve its delivery or performance.

While promising, the current study is constrained by a small sample size and the absence of mechanistic confirmation of OCTN1 involvement. In future studies, we will focus on refining optimal dosages, exploring mechanisms using molecular markers, evaluating systemic absorption, and validating long-term efficacy in human skin models.

## Figures and Tables

**Table 1:** The values of parameters of the control group, EGT at concentration of 130 and 650 µg/cm^2^ using the Logistic model.

Parameters	Control	EGT 130	EGT 650
L	69.7	94.2	94.2
k	0.61	0.34	0.33
x_0_	9.03	7.21	7.17
s≈	11.27	7.13	6.73
ν	45	43	40

s≈: Residual standard deviation (biological variability between animals) ν: Degrees of freedom.

## Data Availability

The original contributions presented in this study are included in the article/Supplementary Material. Further inquiries can be directed to the corresponding author.

## Supplementary Materials

The following supporting information can be downloaded at: https://www.mdpi.com/article/10.3390/ijms27146102/s1.
