# Supplementary material for: Unveiling and Benefits of Topically Applied l-(+)-Ergothioneine in Periwound Region
Source: Int J Mol Sci. 2026 Jul 8;27(14):6102. doi: 10.3390/ijms27146102 (PMC13410314; doi:10.3390/ijms27146102)
Supplement: Supplementary file 1 [file ijms-27-06102-s001.zip › ijms-4327939-supplementary.pdf]

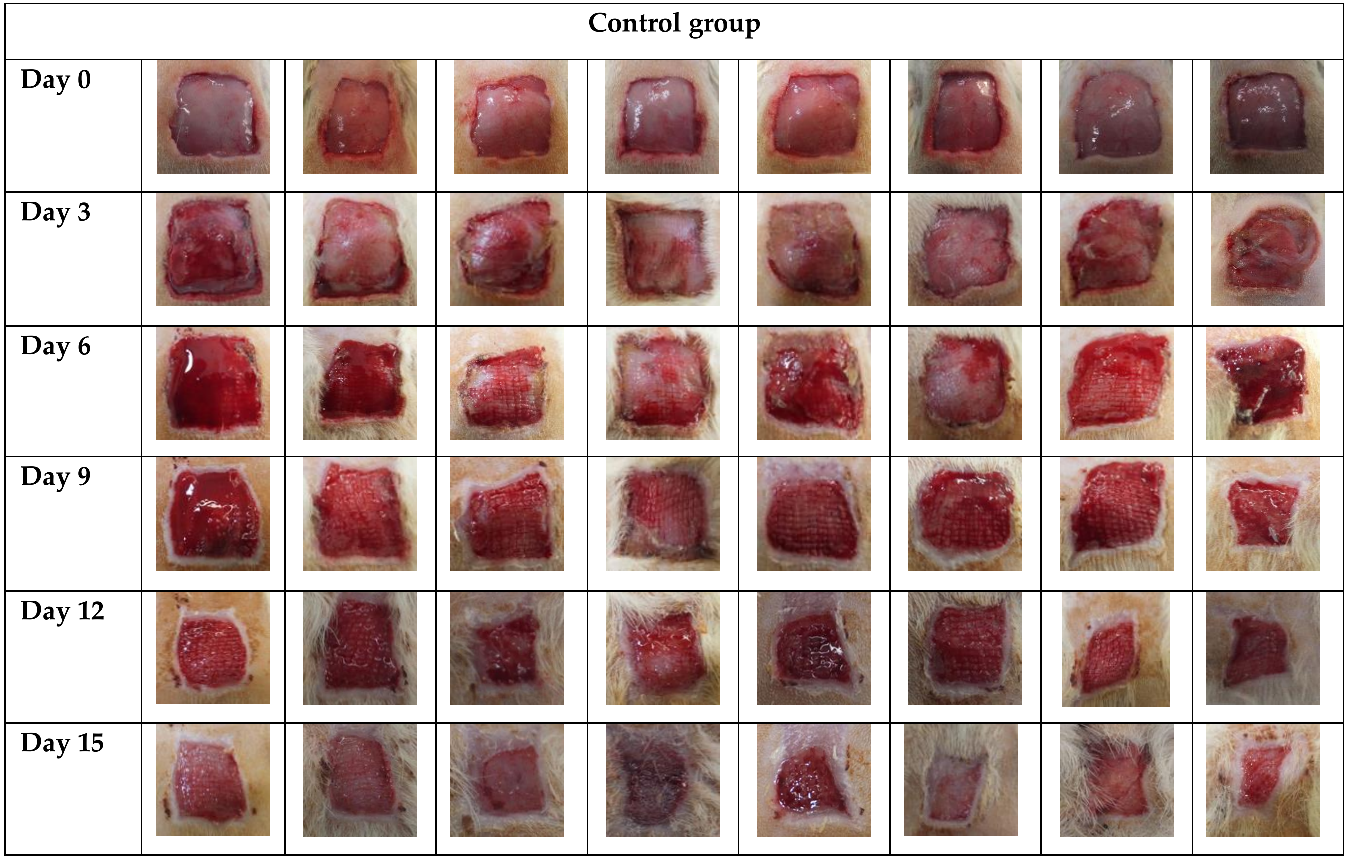

Figure S1. Skin wounds in the control group of rats on days 0, 3, 6, 9, 12 and 15.

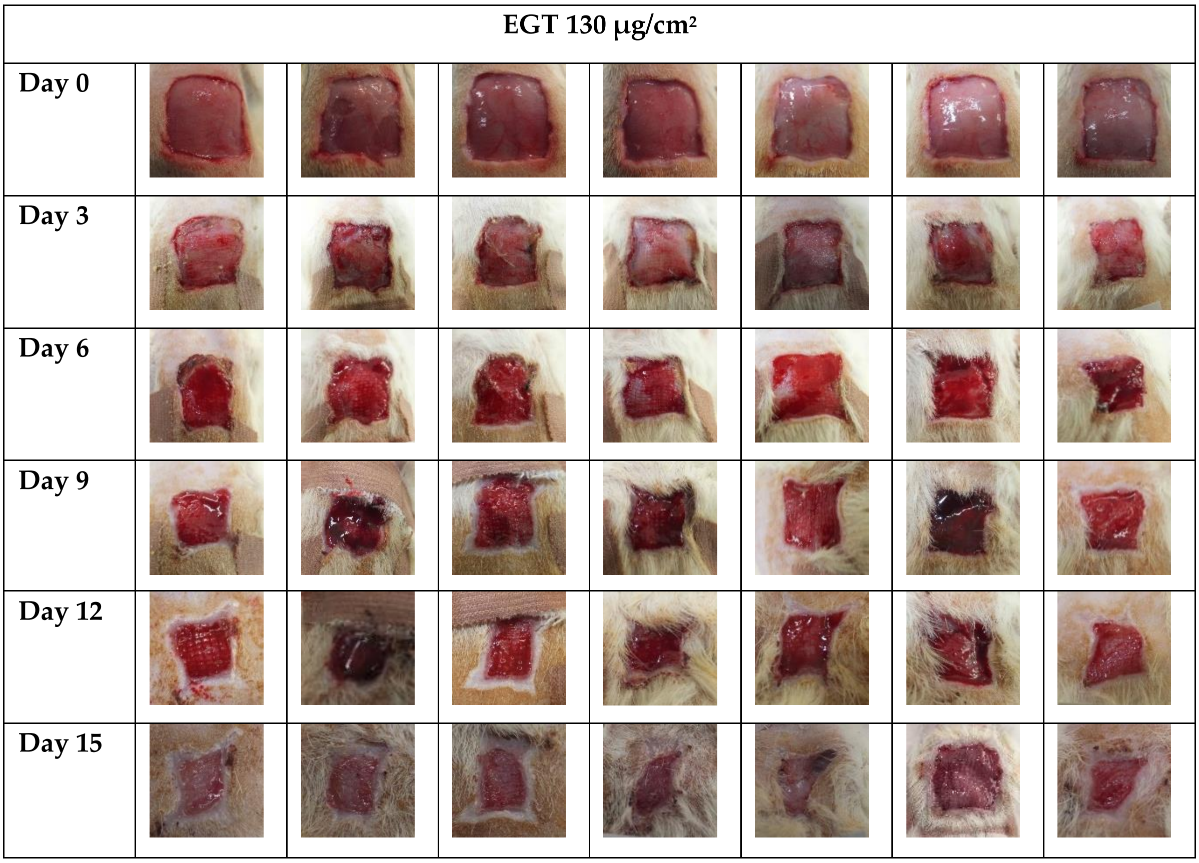

Figure S2. Skin wounds in rats treated with 130 μg/cm² EGT on days 0, 3, 6, 9, 12 and 15.

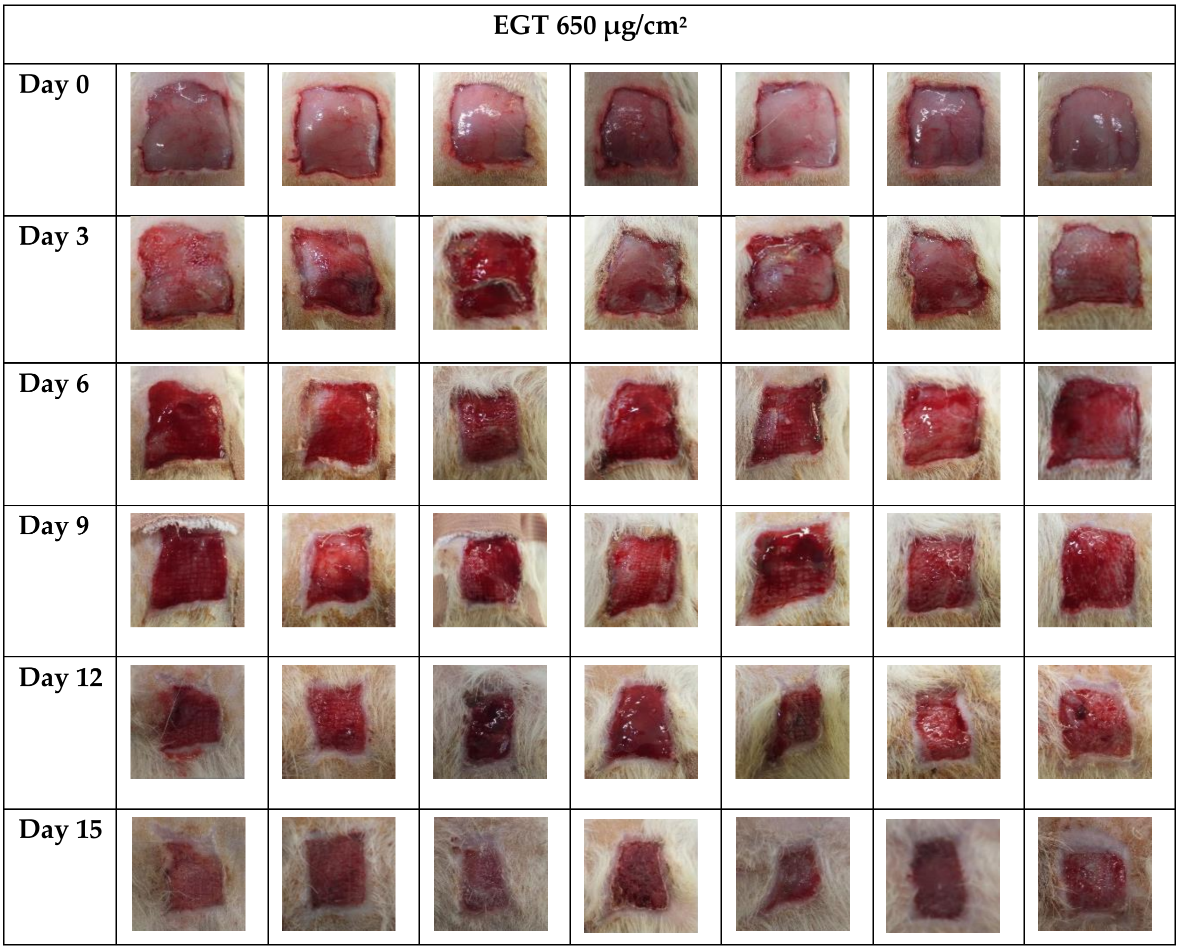

Figure S3. Skin wounds in rats treated with 650 µg/cm² EGT on days 0, 3, 6, 9, 12 and 15.

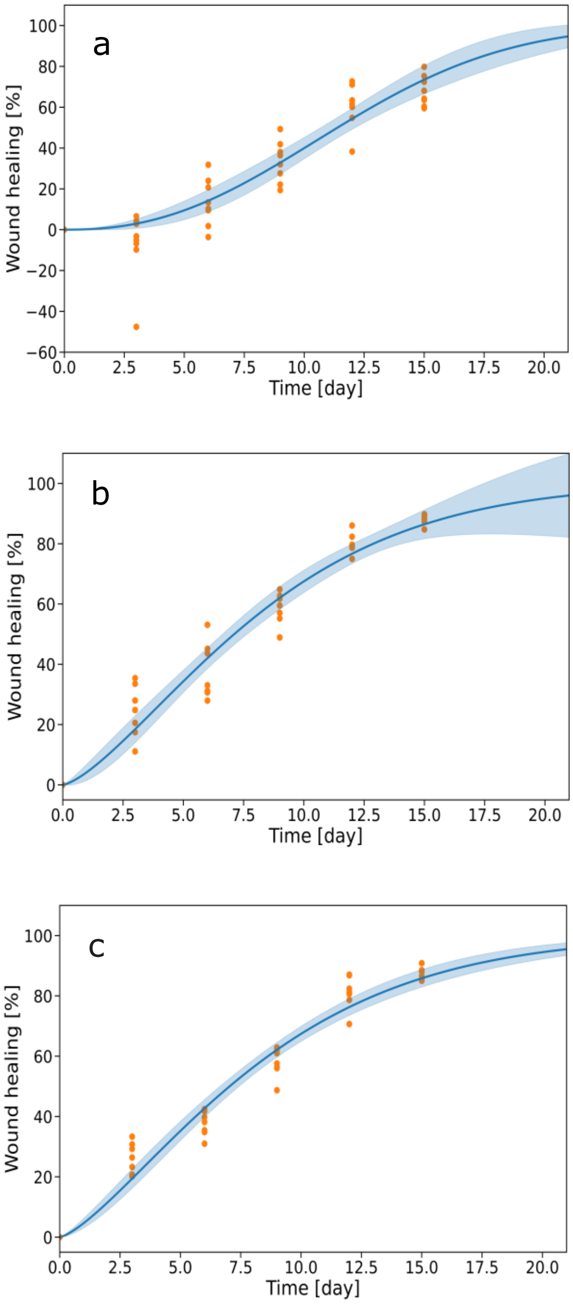

Figure S4 a). The Weibull saturated model estimating the kinetics of skin wound healing in the untreated rats, b) in rats treated with L-(+)-ergothioneine at concentration 130 $\mu g/cm^2$, c) in rats treated with L-(+)-ergothioneine at concentration 650 $\mu g/cm^2$; over 15 days. The blue area expresses 95% interval of confidence.

Similarly to the Logistic saturated model, Weibull saturated model estimated wound healing in the control group reached 91.5% on day 21 [Figure S4a)]. In Figure S4b) Weibull model gradually grows towards a plateau. Since the data do not yet reach clear saturation, on day 21 the parameter L is estimated to be very high (over 100%).

Similar result, although with lower estimated parameter L, was observed in Figure S4c) when examining EGT at concentration of 650 $\mu g/cm^2$. In all figures the value $m > 1$ (Table S1) points out to a significant accelerating phase typical for the proliferative phase of healing.

Table S1. The values of the parameters of the control group, the rats treated with L-(+)-ergothioneine at concentration of 130 and 650 μg/cm^2 using the Weibull saturated model (max. healing 100%).

Parameters	Control	EGT 130	EGT 650
L	100	100	100
t	13.3	9.21	9.20
m	2.36	1.41	1.37
ν	46	41	40

L: Maximal effect of healing equals to 100%
t: Time scale (when the effect begins to appear)
m: Steepness (how sharply the curve breaks)
ν: Degrees of freedom

Table S1 shows data of the analysis of the skin wound healing in the control group, the rats treated with EGT at concentration of 130 and 650 μg/cm^2 using the Weibull saturated model, maximal healing 100%; meaning that the treatments were evaluated relative to a theoretical full recovery. The parameter t, which reflects the characteristic time to reach saturation, is notably higher in the control group (13.3) than in EGT 130 (9.21) and EGT 650 (9.20). This indicates that both EGT treatments accelerate the healing process compared to the untreated control. The shape parameter m is also higher in the control group (2.36) than in EGT 130 (1.41) and EGT 650 (1.37). A higher m value suggests a steeper and more delayed rise, whereas lower values in the EGT treatments indicate a more gradual and earlier onset of the healing response.

Concluding, the results of the Weibull model show that EGT 130 and EGT 650 substantially shortened the time required to reach maximum healing and modify the healing dynamics compared to the control group.

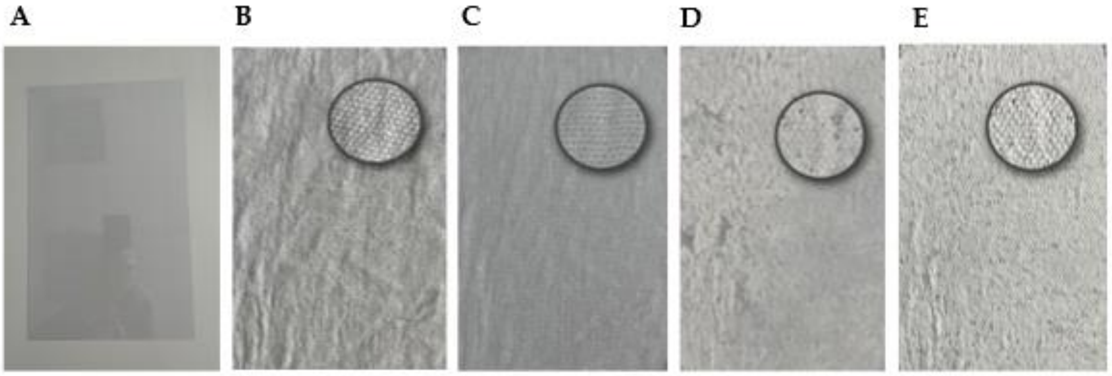

Figure S5. A – polyterephthalate plastic pad, B – gauze, C – gauze impregnated with EGT solution, D – frozen gauze impregnated with EGT, E – lyophilized gauze impregnated with EGT.

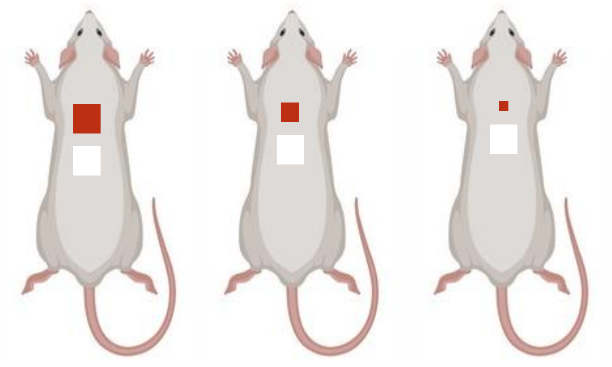

Figure S6. Scheme of placing a gauze in the periwound region with the illustration of the wound healing progress, red square: skin wound, white square: gauze.

The Egyptian cotton is world-wide known natural material possessing an excellent quality. It is *in vivo* composed of long fibers that, after processing, predicts the material to be very soft, extremely absorbable, breathable, superlatively smooth and long-lasting, moreover, it exhibits an extraordinary stability (Figure S7). The addition of L-(+)-ergothioneine even after mega-loading (650 μg/cm^2) into the gauze allows for sustained release of L-(+)-ergothioneine into the periwound region.

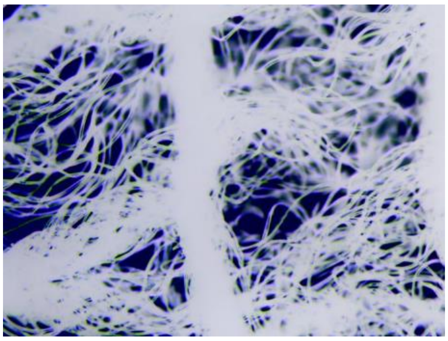

Figure S7. Microfibers of the Egyptian cotton gauze used in advantage in our study (Tri M Medical, Cairo, Egypt).
